# Data on protein changes of chick vitreous during normal eye growth using data-independent acquisition (SWATH-MS)

**DOI:** 10.1016/j.dib.2020.105576

**Published:** 2020-04-18

**Authors:** Jimmy Ka-Wai Cheung, King-Kit Li, Lei Zhou, Chi-Ho To, Thomas Chuen Lam

**Affiliations:** a Laboratory of Experimental Optometry, Centre for Myopia Research, School of Optometry, The Hong Kong Polytechnic University, Hong Kong Special Administractive Region of China; b Ocular Proteomics Laboratory, Singapore Eye Research Institute, Singapore; c Department of Ophthalmology, Yong Loo Lin School of Medicine, National University of Singapore, Singapore; d Ophthalmology and Visual Sciences Academic Clinical Research Program, Duke-NUS Medical School, National University of Singapore, Singapore

**Keywords:** Vitreous, SWATH, Emmetropization, Chick

## Abstract

Myopia is the most common refractive error which is estimated to affect half the population of the world by 2050. It has been suggested that it could be determined by multiple factors such as environmental and genetic, but the mechanism behind the cause of myopia is still yet to be identified. Vitreous humor (VH) is a transparent gelatin-like substance that takes up to 80% of the volume of the eye, making it the largest component of the eye. Although VH is the main contributor to axial elongation of the eye including normal eye growth (emmetropization) and myopia, the diluted nature of VH (made up of 99% of water) made it difficult for less abundant molecules to be identified and therefore often overlooked. Using the more sensitive label-free mass spectrometry approach with data-independent acquisition (SWATH-MS), we established a comprehensive VH proteome library in chick animal model and quantified possible protein biomarkers that are responsible for the axial elongation during emmetropization (7, 14, 21, 28 days after hatching, *n* = 48 eyes). Raw data files for both information-dependent acquisition (IDA) and data-independent acquisition (SWATH-MS) were uploaded on PeptideAtlas for public access (http://www.peptideatlas.org/PASS/PASS01258).

Specifications table

**Table:** 

Subject	Biology
Specific subject area	Normal eye development of chick vitreous
Type of data	Table, Graph, Figure
How data were acquired	Information-dependent acquisition (IDA) and Data-independent acquisition (SWATH-MS) using TripleTOF (QTOF) 6600 mass spectrometer (SCIEX)
Data format	Raw and Analyzed
Parameters for data collection	Vitreous proteins were extracted during normal growth (7, 14, 21 and 28 days old, *n* = 12 eyes at each time point). Digested vitreous peptides were pooled as Right eye pool (RE) and Left eye pool (LE) for each time point and were used for protein identification and quantification using data-independent acquisition (SWATH-MS)
Description of data collection	Information-dependent acquisition (IDA) followed by database search against Gallus reference proteome from UniProt (organism ID: 9031)
Data source location	Centre for Myopia Research, School of Optometry, The Hong Kong Polytechnic University, Hong Kong Special Administrative Region of China
Data accessibility	Repository name: Peptide Atlas repository
	Data identification number: PASS01258
	Direct URL to data: http://www.peptideatlas.org/PASS/PASS01258

## Value of the data

• This dataset is the first report of temporal chick vitreous proteomes available to cover the sensitive period of normal ocular growth (across time points of Day 7, 14, 21 and 28).
• Proteomes of this period could provide new insights to the development of myopia as it is at the early stage of axial elongation within vitreous.
• This data provides novel growth-related proteins in response to the emmetropization and myopia mechanism which could lead to potential myopia-related biomarkers.

## 1 Data description

The emmetropization period describes the stage where the eye elongates from a shorter stage (hyperopic) towards normal refraction stage when the image perfectly aligns to the retina. This is common in humans [1] and animal species [2,3]. For chicks, it takes at least a month of age to complete the emmetropization process. Myopia is considered as a failure of emmetropization where the eyeball further elongates passing the emmetropization state with an indefinite stop sign as it progresses. Therefore, studying this emmetropization period could bring us new insights in the early onset of myopia development mechanism.

**Fig. 1:**
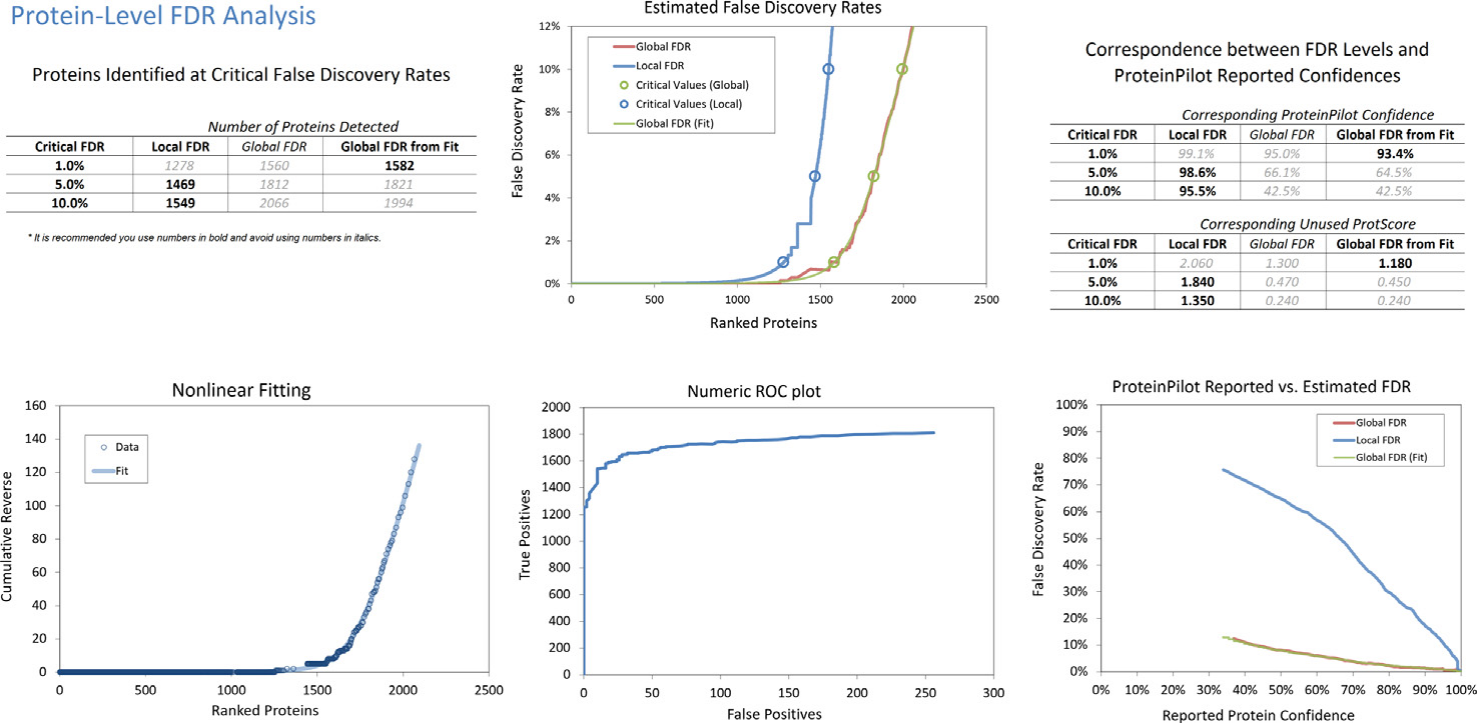
FDR analysis report of comprehensive IDA library (combined search from all time points) from chicken vitreous at protein level generated by ProteinPilot™ software. Top left: A summary of the number of proteins identified under different FDR settings (1%, 5% and 10% for both local and global). Bottom left: A nonlinear flitting plot showing the nonlinear curve fitting for global FDR calculation. Top middle: Estimated false discovery rates plot to show the error rates at all thresholds for both global and local FDR calculation. Circle symbol indicates the different FDR setting of the proteins (1%, 5% and 10% for both local and global). Bottom middle: A numeric receiver operating characteristic (ROC) plot showing the tradeoff between the true identifications that can be obtained vs the number of false positive identifications that needed to be tolerated. Top and bottom right: A table and plot to show the comparison of the confidence reported in ProteinPilot™ software results to the error rates determined by the FDR analysis (local and global).

**Fig. 2:**
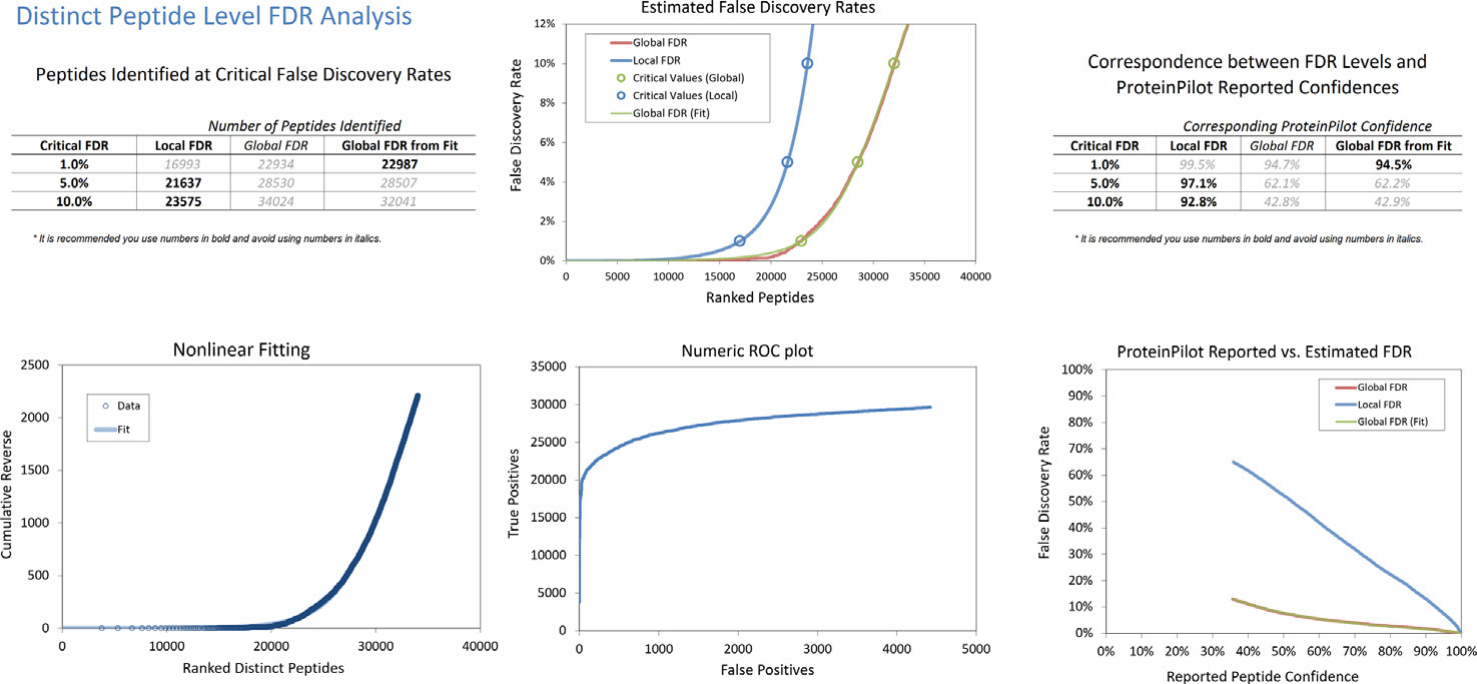
FDR analysis report of comprehensive IDA library (combined search from all time points) from chicken vitreous at peptide level generated by ProteinPilot software. Top left: A summary of the number of peptides identified under different FDR settings (1%, 5% and 10% for both local and global). Bottom left: A nonlinear flitting plot showing the nonlinear curve fitting for global FDR calculation. Top middle: Estimated false discovery rates plot to show the error rates at all thresholds for both global and local FDR calculation. Circle symbol indicates the different FDR setting of the peptides (1%, 5% and 10% for both local and global). Bottom middle: A numeric receiver operating characteristic (ROC) plot showing the tradeoff between the true identifications that can be obtained vs the number of false positive identifications that needed to be tolerated. Top and bottom right: A table and plot to show the comparison of the confidence reported in ProteinPilot™ software results to the error rates determined by the FDR analysis (local and global).

The protein expression in chick vitreous during normal growth (emmetropization) period was identified and quantified using data-independent acquisition (SWATH-MS). Acquired data by information-dependent acquisition (IDA) was combined and searched against the UniProt database (Gallus gallus 42,584) for protein identification using ProteinPilot (v5.0, SCIEX) [4]. The protein and peptide analysis at 1% FDR are shown in Fig. 1, Fig. 2, respectively. At 1% FDR, 1582 non-redundant proteins and 22,987 distinct peptides were generated from the combined ion library (a total of 8 raw injections) for SWATH-MS quantification (Table S1). Top ten most significant differentially expressed proteins across all the time points quantified by SWATH-MS are listed in Table 1 (proteins with ≥ 1.5-fold change, ≥2 peptides per protein and a fold change (FC) expression that must be the same direction for both eyes).

## 2 Experimental design, materials, and methods

### 2.1 Chick vitreous collection

Both eyes of 6 White leghorn chicks (Gallus gallus domesticus) during emmetropization (Day 7, 14, 21, 28) were used throughout the experiments (*n* = 48 eyes). In-house hatched chicks were raised in stainless steel brooders under a 12/12 dark/ light cycle with an average luminance of 500 lx at the center of the cages. Refractive error was measured using a streak retinoscope (Heine, Beta 200 Streak Retinoscope) and ocular components measurements were measured using an A-Scan Ultrasound (Olympus 5073PR and Watson Marlow 505 u pump system) coupled with a 30 MHz probe (PZ25-025-R1.00) to ensure normal eye growth during the study period. The breeding room has an automatically controlled temperature environment with an average temperature of 25.8 °C and a humidity level of 41.8% during the study period. All chicks were given Food and water ad libitum. Handling and operations throughout the experiment were in compliance with the ARVO statement and approved by the Hong Kong Polytechnic University Animal Subjects Ethics Sub-committee (ASESC).

### 2.2 Vitreous protein extraction

Chicks at each time point were sacrificed with carbon dioxide asphyxiation. The optic nerve was cut off immediately to allow the collection of the eyeball. The eyeball was then transferred into ice-cold phosphate-buffered saline (PBS) and the muscle tissues around the eyeball were carefully dissected. The eyeball was then hemisected equatorially using a razor and the vitreous body was extracted and stored in a 1.5 mL Eppendorf tube while the remains of retina and retinal pigment epithelium (RPE) were removed carefully. The vitreous body was then weighted and kept in frozen liquid nitrogen for homogenization. A 1:1 w/v ratio of T-PER buffer (Thermo Fisher Scientific, U.S.) was added to the each collected vitreous sample and were homogenized using the homogenizer (Precellys Evolution, Bertin Instrument) with the following settings: 5000 rpm for 30 s with 4 cycles under liquid nitrogen cooled environment. The protein concentration of homogenized vitreous samples was then measured by Bradford protein assay (Bio-Rad, U.S.).

### 2.3 Vitreous sample preparation

Equal protein amounts (15 µg) from each sample (*n* = 6) were reduced with 10 mM dithiothreitol (DTT) for 1 h at 37 °C. For alkylation, 40 mM of iodoacetamide (IAA) was added into the sample and incubated in the dark for 30 mins at room temperature. Vitreous samples were then added with ice-cold acetone overnight at −20 °C for acetone. After centrifuging at 21,380 x g for 30 mins at 4 °C, 1 M Urea in 25 mM ammonium bicarbonate was used to dissolve the dried protein pellet. Then, trypsin (Promega, U.S.) was added for tryptic digestion at 1:25 (enzyme to substrate ratio, w/w) overnight at 37 °C. Digested samples were cleaned-up using Oasis HLB solid-phase extraction column (Waters, U.S.). Pierce Quantitative Colorimetric Peptide Assay (Thermo Fisher Scientific, U.S.) was used for measuring peptide concentration before MS injection. For each time point, 6 eyes were then pooled as Right eye pool (RE) and Left eye pool (LE). The final peptide concentration was set as 1.2 µg/ µl in 0.1% FA for MS injection.

### 2.4 LC-MS/MS analyses

Mass spectrometry analyses (both IDA and SWATH-MS) were performed on a Triple TOF 6600 mass spectrometer (SCIEX) coupled to an Eksigent 415 nano-LC system. Equal amounts (1.2 µg) of digested peptides were loaded onto the trap column (350 µm x 0.5 mm, C18) at a flow rate of 2 µL min^−1^ for 15 mins and was then separated with a nano-LC column (100 µm x 30 cm, C18) at a flow rate of 350 µL min^−1^. Mobile phase A was a mixture of 0.1% formic acid (v/v), 5% acetonitrile (v/v) in water and mobile phase B contains 0.1% formic acid(v/v), 98% acetonitrile (v/v) in water. The gradient settings and conditions used are described in our previous study [5], in brief: 0–0.5 min: 5%B, 0.5–90 min:10%B, 90–120 min:20%B, 120–130 min:28%B, 130–135 min:45%B, 135–141 min:80%B, 141–155 min:5%B. The eluent was introduced into the TripleTOF 6600 system with a 10 µm SilicaTip electrospray emitter. The TOF-MS survey scan range was set between 350 *m/z*-1800 *m/z* (250 ms accumulation time), then MS/MS scans from 100 *m/z*- 1800 *m/z* (50 ms accumulation time) in high sensitivity mode with rolling collision energy for collision induced dissociation followed. SWATH-MS was acquired with 100 variable isolation windows between the mass range of 100 *m/z*- 1800 *m/z* (29 ms accumulation time) resulting in a total cycle time of 3.0 s and each sample was performed in triplicate.

### 2.5 Generation of ion library and SWATH-MS quantification

Tryptic digested peptides from RE and LE at each selected time point (8 separate IDA injections) were combined for the generation of the combined ion library (.group). Database (.fasta) was downloaded from UniProt (Gallus gallus 42,584) and matched with the acquired data using ProteinPilot 5.0 software with a 1% false discovery rate (FDR) setting for protein identification (ID). Three technical DIA (SWATH-MS) injections were acquired for each POOL sample at each condition (Day 7, 14, 21, 28), having a total of 24 total injections. Acquired SWATH-MS raw data (.wiff) were then processed in PeakView (v2.1, SCIEX). The processed data were then exported to MarkerView (v1.3.1, SCIEX) and MLR normalization method [6] was selected for SWATH-MS statistical analysis. Only proteins with ≥ 2 peptides per protein, ≥ 1.5-fold change, FC expression must be the same direction for both eyes were classified to be differentially expressed.

## Appendix Supplementary materials

Image, application 1

Image, application 2
